# Supplementary material for: Features of Nuclear Export Signals of NS2 Protein of Influenza D Virus
Source: Viruses. 2020 Sep 29;12(10):1100. doi: 10.3390/v12101100 (PMC7600798; doi:10.3390/v12101100)
Supplement: Supplementary file 1 [file viruses-12-01100-s001.pdf]

viruses

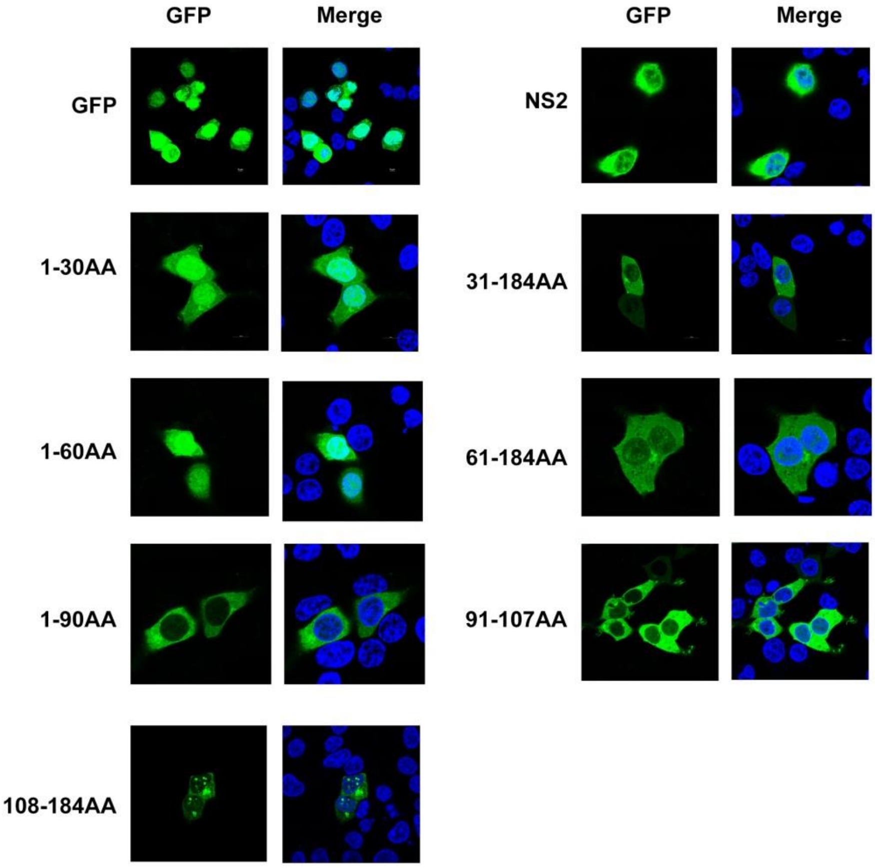

Figure S1. HEK293T cells were transfected with truncated constructs (1–30aa, 31–184aa, 1–60aa, 61–184aa, 1–90aa, 91–107aa, and 108–184aa) corresponding to Figure 3. Cells were then fixed, permeabilized, and stained with the 4, 6-diamidino-2-phenylindole (DAPI) and subcellular localization of eGFP fusion protein were examined via fluorescence microscopy 24 h post-transfection.

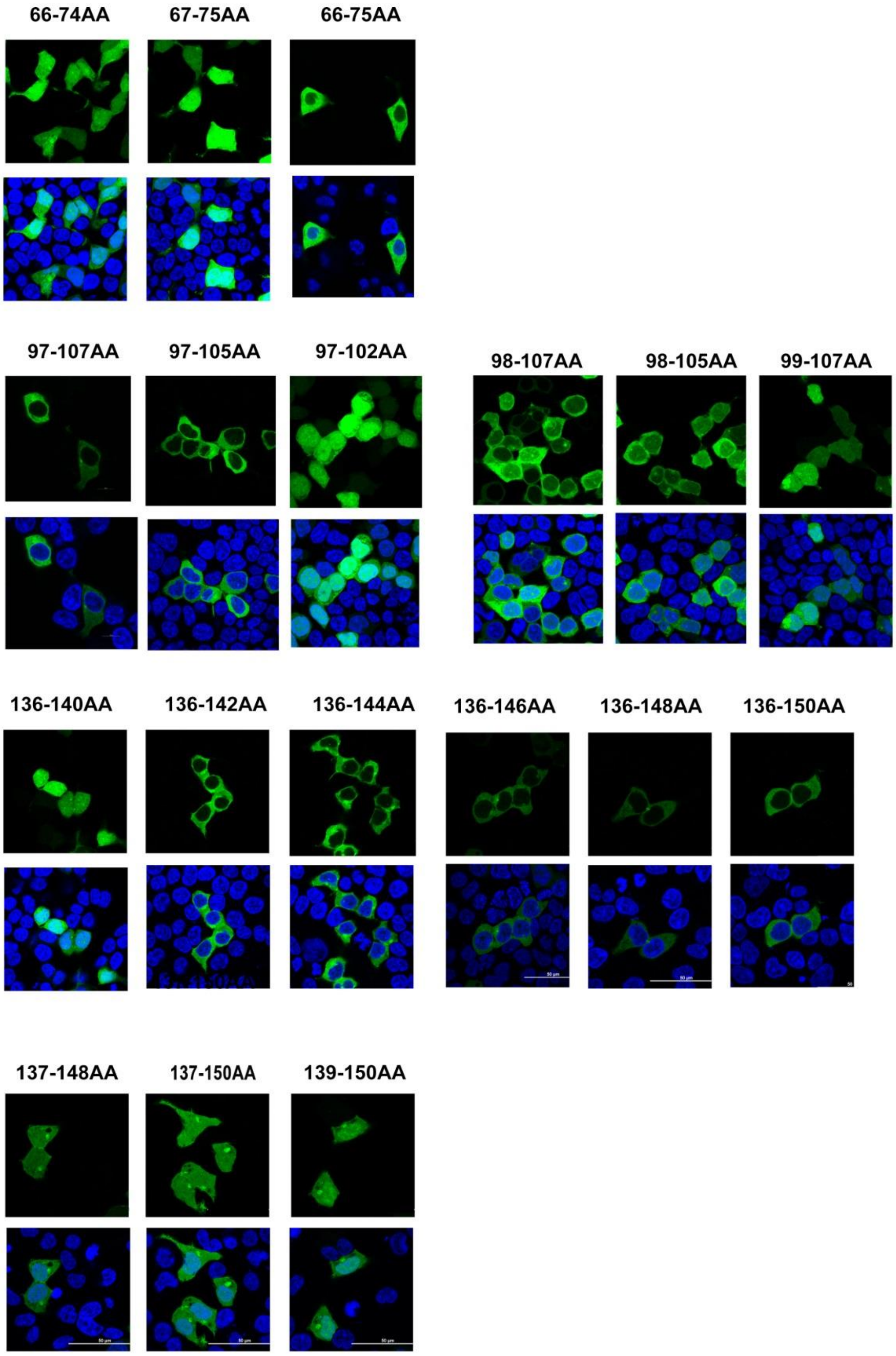

Figure S2. pCAGGS-eGFP-NES were transfected into HEK293T cells corresponding to Figure 4. Cells were then fixed, permeabilized, and stained with the 4, 6-diamidino-2-phenylindole (DAPI) and subcellular localization of eGFP fusion protein were examined via fluorescence microscopy 24 h post-transfection.

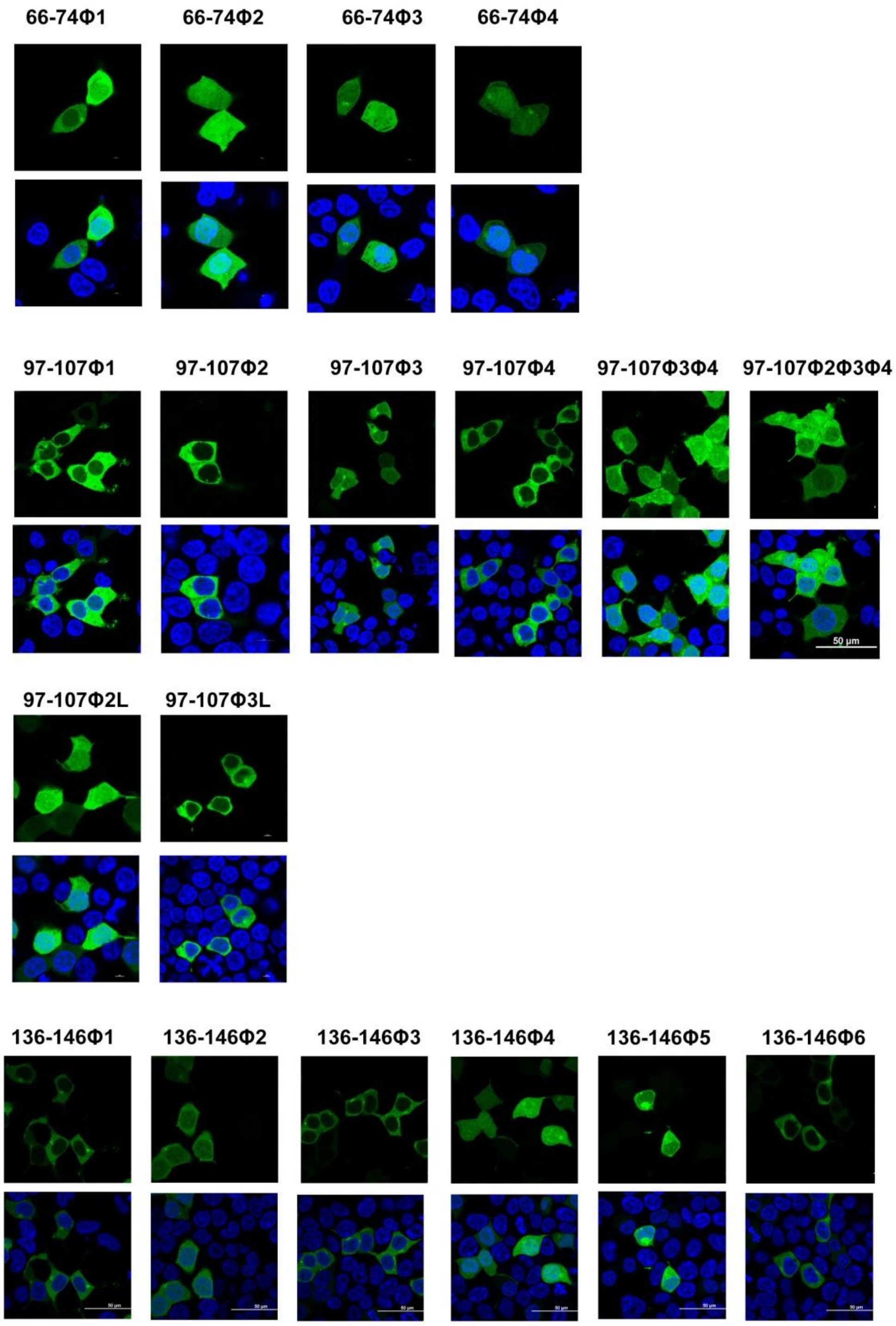

Figure S3. a series of mutant constructions were transfected into HEK293T cells corresponding to Figure 5. Cells were then fixed, permeabilized, and stained with the 4, 6-diamidino-2-phenylindole (DAPI) and subcellular localization of eGFP fusion protein were examined via fluorescence microscopy 24 h post-transfection.

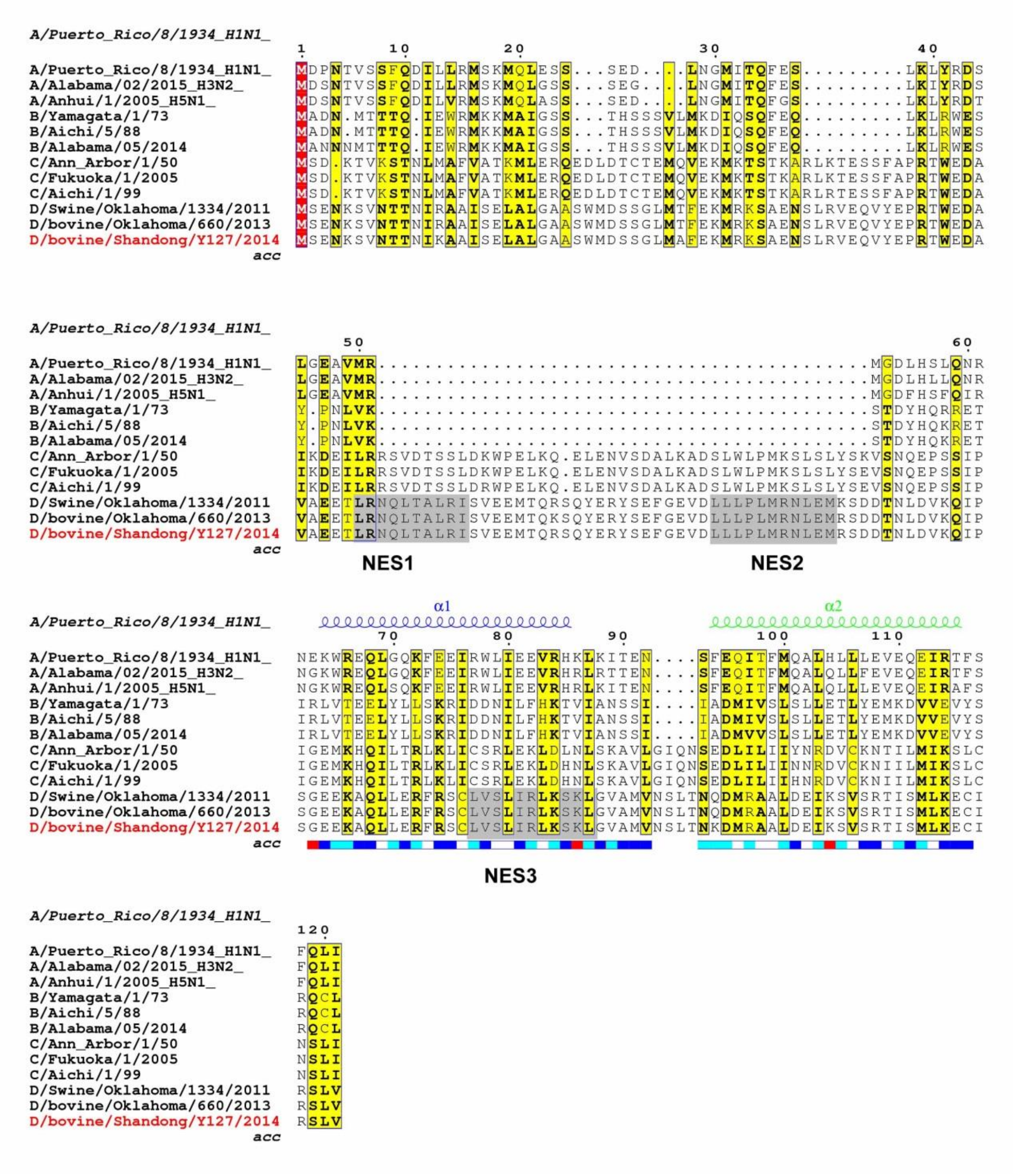

Figure S4. Sequence alignment of NS2 proteins of type A, B, C and D influenza viruses. All sequences were aligned with ClustalW, and the secondary structure elements are defined based on PDB file 1PD3(A/Puerto Rico/8/1934, H1N1) using online program, ESPript server [30]. All the sequences were obtained from the NIAID Influenza Research Database (IRD) online through the web site at http://www.fludb.org.

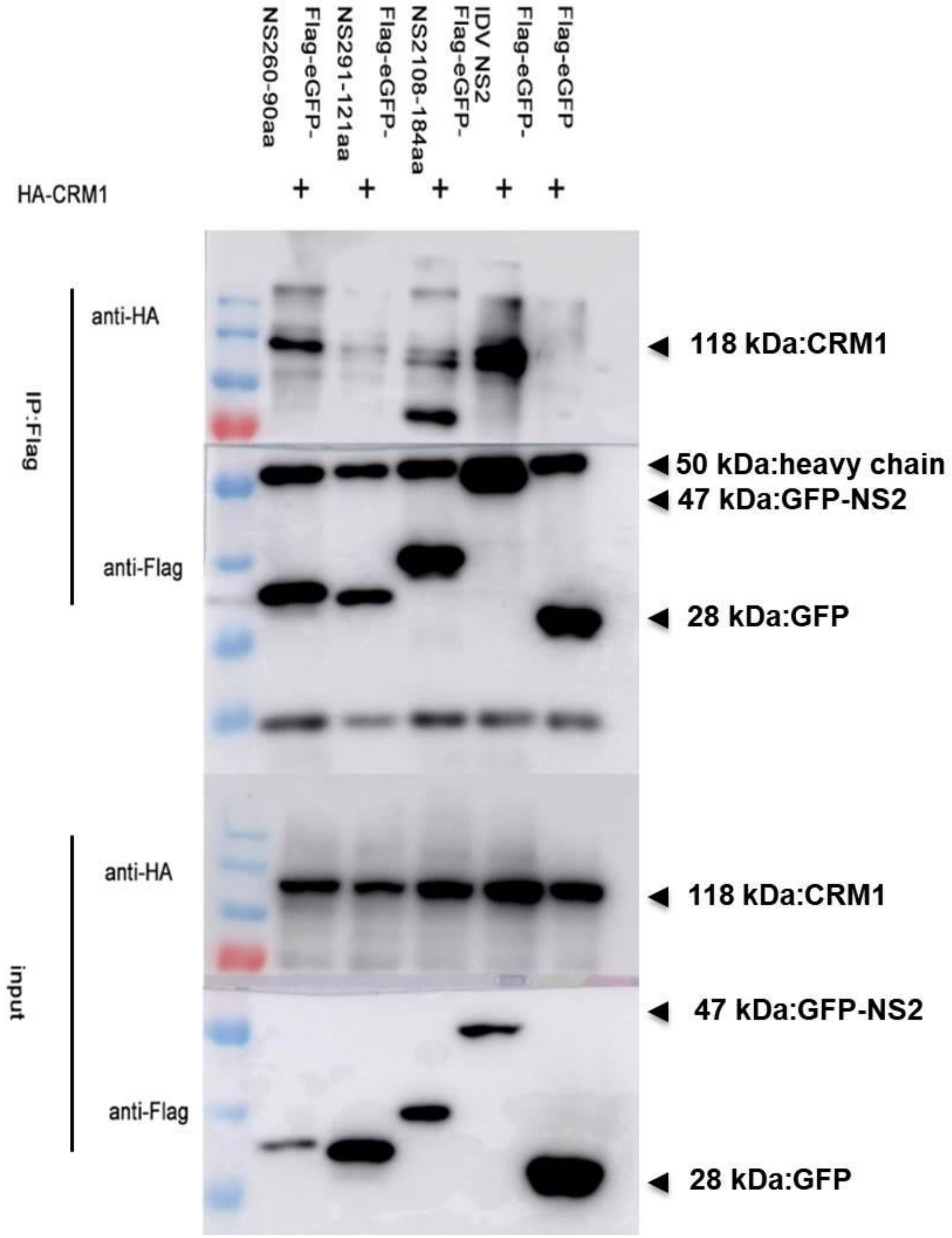

Figure S5. Original image of Figure 5 D.

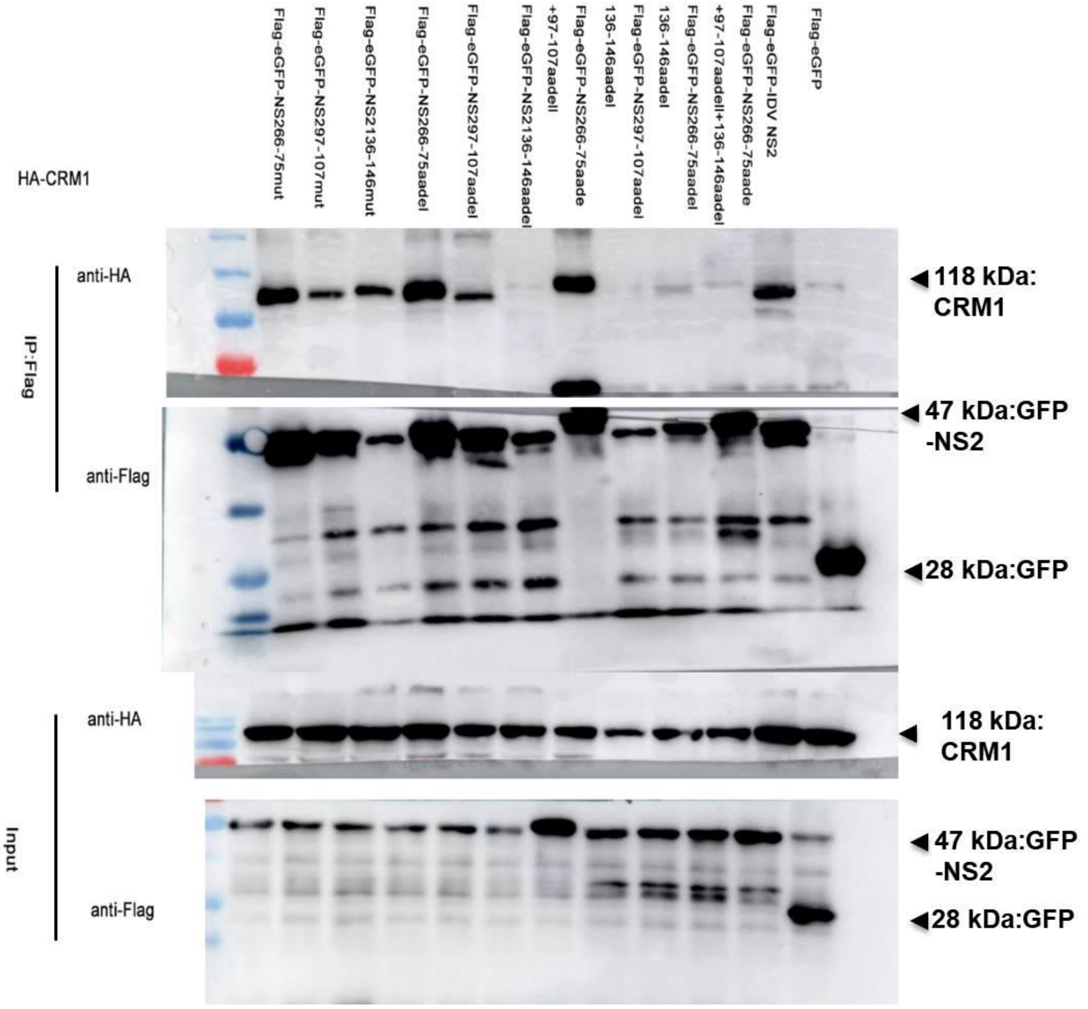

Figure S6. Original image of Figure 6 B.
